# On the rapid use of verb-control information in sentence processing

**DOI:** 10.3389/fpsyg.2023.1320966

**Published:** 2024-01-04

**Authors:** Josep Demestre

**Affiliations:** Departament de Psicologia and CRAMC, Universitat Rovira i Virgili, Tarragona, Spain

**Keywords:** parsing, control information, null subject, antecedent assignment, Spanish

## Abstract

A central topic in psycholinguistics is the study of how and when the parser assigns an antecedent to referentially-dependent elements. One such referentially-dependent element is the null subject of non-finite clauses. The aim of the present study was to examine the role of verb control information in the assignment of an antecedent to such a null subject. The results so far are inconclusive. Some authors argue that verb control information has a late influence, whereas others argue that such verb-specific information has a very rapid influence. We report a self-paced reading study in Spanish in which verb type (subject vs. object control) and grammaticality (grammatical vs. ungrammatical) were manipulated. The grammaticality manipulation was carried out by introducing a person anomaly at the infinitive itself, and not at a later word (e.g., “Te prometí/aconsejé adelgazarme/adelgazarte cinco quilos en un mes.” Literal translation, “I to you promised/advised to lose_myself/yourself_ five kilos in a month”). With such a manipulation we can examine whether at the first possible point (i.e., the infinitive) verb control information was used to assign the correct antecedent (i.e., the subject in sentences with a subject-control verb, and the object in sentences with an object-control verb) to PRO. The results showed that at the infinitive there was a main effect of grammaticality, meaning that the correct antecedent has already been assigned to PRO. The present findings are consistent with models that assume that verb-specific information plays an important role in the initial stages of sentence processing.

## 1 Introduction

A central issue in the study of online language comprehension is how and when the parser assigns an antecedent to referentially-dependent elements; natural language is full of referentially-dependent elements, whose referents must be determined in order to arrive at a coherent interpretation of the linguistic message. The task of assigning an antecedent to referentially-dependent elements is complex since the parser often has to use different sources of information (e.g., lexico-semantic, syntactic, discourse information). One question that has to be answered is whether antecedent assignment processes are initiated immediately upon perception of an anaphoric expression or whether such processes are delayed.

One referentially-dependent element is the subject of nonfinite clauses. The subject of a nonfinite clause has no phonological content (i.e., it is null) and must therefore be understood implicitly. In generative linguistics (Chomsky, 1986), the null subject of a nonfinite clause is called PRO (or “big PRO,” distinct from *pro*^1^ or “small pro”). In obligatory control constructions, PRO inherits the minimal identifying features from another NP with which it is coindexed. This coreference is determined by a relationship called *control* (Chomsky, 1981; Manzini, 1983). When PRO appears in an infinitival complement clause, one of the arguments of the matrix verb will be understood as its antecedent (or controller). Whether the controller is the subject or the object of the matrix clause depends on the intrinsic lexical properties of this verb. In (1), the reflexive in the embedded clause takes as its antecedent the controlling NP. The ungrammatical reflexives show that *promise* is a subject control (SC) verb and *persuade* is an object control (OC) verb.

(1)a.Mary_i_ promised Bill_j_ *PRO*_i_ to feed herself/*himself.
b.Mary_i_ persuaded Bill_j_ *PRO*_j_ to feed himself/*herself.

To correctly coindex PRO with its antecedent, the parser must likewise use information specific to the main verb. However, the availability of information in the stimulus does not necessarily mean that this information is immediately accessible to the language processor. The question is not if, but when the processor uses this information.

Control has been at the center of syntactic theorizing since its introduction into the generative literature. In the theoretical linguistics literature, there are two opposing approaches to control. On one hand, there are theories that assume that control involves primarily syntactic factors (e.g., Rosenbaum, 1967; Hornstein, 1999). Rosenbaum proposed that control is constrained by the Minimal Distance Principle, a locality principle that states that the controller of the null subject must be the closest NP in the syntactic tree. According to Hornstein, control is derived by movement, a purely syntactic operation. Minimalist approaches propose the Minimal Link Condition (Chomsky and Lasnik, 2003), that assumes that each step in the derivation should be minimal and, thus, shorter derivations are preferred over longer ones. On the other hand, another tradition assumes that most of the factors involved in control are semantic. Such theories focuses on the importance of the lexical-semantics of the predicate that selects the infinitival complement (e.g., Jackendoff and Culicover, 2003; Culicover and Jackendoff, 2006). According to such theories, the type of control (i.e., SC or OC) a complement displays is determined by the thematic roles the main verb assigns to its arguments.

The present study aims to examine the early or late influence of verb control information on the assignment of an antecedent to the null subject of an infinitival complement clause.^2^ More specifically, we want to answer the question of whether control information is made available to and used by the parser as soon as the infinitive verb is recognized. Before going into the details of the study, we will review the existing experimental literature on the role of verb control information in the assignment of an antecedent to PRO.

The existing empirical evidence on the influence of control information in the processing of PRO has shown mixed results: while some authors have argued that control information is not accessed in early parsing stages, others have claimed that control information is accessed and used from very early on. The evidence in favor of each of these two opposing proposals will be examined in turn.

A seminal study on the processing of empty categories is that by Frazier et al. (1983) and Clifton and Frazier (1986), who used an end-of-sentence (i.e., offline) comprehension task. The authors concluded that control information does not constrain initial antecedent selection. According to the authors, control information is used only after an initial heuristically guided assignment of fillers to gaps has been made (Clifton and Frazier, 1986). This heuristic, called “the most recent filler strategy,” claims that the less distant NP that occurs in a potential filler position in the phrase-marker is selected as the antecedent of PRO.^3^ The most recent filler strategy predicts that an initial dependency is formed between PRO and the most recent potential antecedent, and this dependency is then checked against control information. In the case of SC-sentences, the initial dependency between PRO and the less distant NP (i.e., NP2) has to be revised once control information is made available. According to this account, this revision would cause difficulties in SC-sentences.

A second seminal study was conducted by Boland et al. (1990), who tested structures similar to those used by Frazier et al. (1983). Rather than using an offline comprehension task, Boland et al. (1990) used an online plausibility monitoring task. The results showed immediate sensitivity to non-plausible continuations at the first point where they could be detected (i.e., the infinitive verb), leading the authors to conclude that control information was used “very early, if not immediately” (Boland et al., 1990).

It is worth noting that both Frazier et al. (1983) and Boland et al. (1990) did not isolate the phenomenon of control from that of movement, as they used sentences containing both a PRO and a wh-trace.^4^ This could lead to a complex interaction between the two types of empty categories, making it difficult to adequately examine the processes involved in assigning an antecedent to PRO.

Demestre et al. (1999) introduced a new way to study the assignment of an antecedent to PRO in Spanish by using gender (dis)agreement between an adjective in the infinitival clause and an NP in the main clause. Such manipulation is possible thanks to the morphological richness of Spanish, a language in which determiners, quantifiers, and adjectives carry overt gender morphology that displays the agreement values inherited from the noun they modify. PRO lacks morphology, but it receives its features from its antecedent. Demestre and colleagues recorded participants’ electroencephalogram (EEG) while listening to OC-sentences in which the matrix clause contained two first names, one in the subject position, and one in the object position. One name was feminine, and the other one was masculine. A clash in gender agreement was created by reversing the syntactic position of the two names. The gender of the adjective in the infinitival clause was kept constant in the two conditions (e.g., the Spanish translation of sentences such as “*Peter^masc^/Mary^fem^ has advised Mary^fem^/Peter^masc^ to be polite^fem”^*). According to the authors, the detection of the grammatical violation at the adjective would imply that the processor had established the coreference relation between PRO and its controller. The results showed that agreement violations elicited a biphasic response (i.e., LAN + P600), showing that the brain reacts immediately to agreement violations, and so it can be assumed that the parser had quickly assigned the correct antecedent to PRO. A limitation of this study is that it only used OC-verbs, and, thus, it cannot provide direct evidence for the early or late influence of control information in assigning an antecedent to PRO. Their results could be explained both by theories that assume a late influence of control information and by theories that assume a very rapid influence of such a verb-specific information. One could go even further and argue that the results can be explained by (hypothetical) models that assume that control information plays no role at all in sentences processing. The parser could assign the correct antecedent to PRO, but such an assignment could be made without consulting any control information, at either early or late stages of processing.

Two studies (Betancort et al., 2006; Demestre and García-Albea, 2007) overcame the limitations of Demestre et al. (1999)’s study by using both SC-and OC-verbs with two potential antecedents in the main clause. In contrast to Frazier et al. (1983) and Boland et al. (1990), these studies isolated the control phenomenon from the movement phenomenon, i.e., they used sentences containing a PRO but no movement-related phenomenon. The rationale of the two studies was very similar, the only difference being methodological. Whereas Betancort et al. used the eye-tracking technique, Demestre & García-Albea conducted an EEG study. Following Demestre et al. (1999), both studies used gender (dis)agreement between an adjective and PRO (and, ultimately, with its controller) to examine the early or late influence of verb control information in the assignment of an antecedent to PRO. The detection of gender agreement violations (in sentences such as “John_i_^masc^ promised Mary_j_^fem^ *PRO*_i_ to be polite^fem^,” or “John_i_^masc^ advised Mary_j_^fem^ *PRO*_j_ to be polite^masc^”) would imply that the processor had established the coreference relation between PRO and its antecedent. If the parser had detected the null subject and coindexed it with its correct antecedent, then one would expect gender agreement violations in both SC-and OC-items to produce a response associated with anomaly detection (i.e., increased fixation times in early measures of the eye-tracking record, or a P600 response in the ERP). On the other hand, if the parser had detected PRO and coindexed it with the most recent NP, then one would expect an interaction between grammaticality and verb type. Whereas for SC-items one would expect an anomaly detection response to grammatical adjectives (since they disagree in gender with the most recent NP), for OC-items one would expect an anomaly detection response to ungrammatical adjectives (since they disagree in gender with the most recent NP).

The results by Betancort and colleagues showed evidence for the rapid detection of gender agreement violations, as participants took longer to read ungrammatical sentences at the adjective and at the spillover regions. The effect of ungrammaticality was observed for both SC-and OC-sentences. These results seem to indicate that the parser quickly selects the correct antecedent in both SC-and OC-sentences, thus providing evidence for the rapid influence of control information. Demestre & García-Albea’s results point in the same direction, showing that ungrammatical adjectives elicited a P600 effect for both SC-and OC-items. The rapid detection of the anomaly indicates that the parser had established the coreference relation between PRO and its antecedent, and that the processor had rapidly consulted control information to select the correct antecedent of PRO. The authors argue that the results are consistent with parsing models that emphasize the rapid influence of verb-specific information on sentence processing (MacDonald et al., 1994; Trueswell and Tanenhaus, 1994, 1995; McRae and Matsuki, 2013).

The results of these two studies seem to indicate that verb control information is rapidly used when the parser assigns an antecedent to PRO. However, it could be argued that their results do not reflect the first stage of parsing, but a later stage, when verb control information is already available to the parser. Both studies manipulated an adjective that was the word immediately following the infinitive (Demestre and García-Albea, 2007) or the second word after the infinitive (Betancort et al., 2006). One could argue that when processing the infinitive, the parser first follows the most recent filler strategy and proceeds to coindex PRO with NP2. At a second stage, the parser quickly checks its initial assignment once verb control information is available. Such a checking process could be completed before the parser encounters the adjective in the input string. To test this alternative hypothesis, it seems necessary to examine what happens when the parser processes the infinitive itself, rather than a word following it. The main aim of the present study is to investigate whether control information is used when processing the infinitive.

## 2 The present study

In the present self-paced reading experiment, we examined the processing of reflexive infinitive verbs in Spanish. Reflexive verbs are used when the direct (or indirect) object of a sentence is the same as the subject. Reflexive verbs are used frequently in Spanish to describe actions that a person does to, for, or from him or herself. Reflexive verbs consist of an infinitive and a reflexive pronoun. The reflexive pronoun is always marked with the same person (1st, 2nd, and 3rd) and number (singular, plural) as the subject of the sentence. In Spanish, the reflexive pronoun is placed before the verb when the reflexive verb is conjugated, but after (and at the end of) the verb when the reflexive verb is in the infinitive.

In the present study, two variables were manipulated in a 2 × 2 within-participants design. The first variable was verb type: SC-and OC-verb. The second variable was grammaticality: grammatical and ungrammatical. Table 1 shows an example of an experimental item in the four experimental conditions.

**Table 1:** Examples of the experimental sentences.

Verb	Grammaticality	Example sentences
SC	Grammatical	(*pro*_i_) Te_j_ prometí adelgazarme_i_ cinco quilos en un mes.
SC	Ungrammatical	(*pro*_i_) Te_j_ prometí adelgazarte_j_ cinco quilos en un mes.
		(I to you promised to lose_myself/*yourself_ five kilos in a month)
OC	Grammatical	(*pro*_i_) Te_j_ aconsejé adelgazarte_j_ cinco quilos en un mes.
OC	Ungrammatical	(*pro*_i_) Te_j_ aconsejé adelgazarme_i_ cinco quilos en un mes.
		(I to you advised to lose_yourself/*myself_ five kilos in a month)

The literal English translations of the example sentences are given in parentheses.

In all experimental sentences, the subject of the main clause was first person singular, and the object was second person singular. The reflexive pronoun attached to the infinitive could be in first person (i.e., *me*, “to me” in English) or in second person (i.e., *te*, “to you” in English). The reflexive pronoun must agree in person with PRO, and, ultimately, with its controller. In the grammatical conditions the controller and the reflexive pronoun agreed in person. In the ungrammatical conditions it was the non-controller NP, and not the controller, the argument that agreed in person with the reflexive pronoun.

The rationale of the study is that for the parser to detect the anomalies in the infinitive region, control information must already be available. If control information has a rapid influence on the assignment of an antecedent to PRO, then one would expect to find a main effect of grammaticality, with longer reading times for ungrammatical sentences as compared to grammatical ones. On the other hand, if the initial assignment of an antecedent to PRO is blind to verb control information but follows the most recent filler strategy, then one would expect an interaction between verb type and grammaticality. For SC-items, one would expect longer reading times for grammatical sentences than for ungrammatical sentences, since in the grammatical condition the first person reflexive pronoun does not agree in person with the second person object NP. For OC-items, one would expect longer reading times for ungrammatical than for grammatical sentences, since in the ungrammatical condition there is a person clash between the first person reflexive pronoun and the second person object NP.

## 3 Method

### 3.1 Participants

Eighty undergraduate Psychology students (64 females; age range: 20–30, *M* = 21.8, SD = 2.9) from the Universitat Rovira i Virgili (Tarragona, Spain) participated in the experiment. Participants received course credit for taking part in the experiment. All participants were native Spanish speakers with normal or corrected-to-normal vision. All participants gave signed informed consent before the experiment. The study was approved by the Ethics Committee of the Universitat Rovira i Virgili (CEIPSA-2021-PR-0024).

### 3.2 Materials

The experimental materials, the datasets and analysis code for this study are publicly available online on the OSF.^5^ The stimulus sentences consisted of a 2 × 2 Latin square design crossing the factors verb (SC vs. OC) and grammaticality (grammatical vs. ungrammatical). The control verbs were selected from “Gramática descriptiva de la lengua Española” (Bosque and Demonte, 1999), a reference book on Spanish grammar. All experimental items were one sentence long and consisted of eight words. The first word was always the Spanish second person singular dative pronoun “te” (“to you” in English), which plays the role of object of the main clause. The second word was the main verb, which was always a first person singular past tense verb, whose subject was a null subject (i.e., *pro*). Given the rich morphology of the Spanish language, it is redundant to make the subject explicit when the verb is unequivocally marked for number and person. As shown in Table 1, the main verb could be an SC-or an OC-verb. The third word was the critical one. It was the contraction of an infinitive reflexive verb and a reflexive pronoun. The reflexive pronoun could be either *me* (first person), or *te* (second person). In the grammatical conditions, the reflexive pronoun agreed in person with the controller of PRO. In the ungrammatical conditions, the reflexive pronoun disagreed in person with the controller of PRO and agreed with the non-controlling NP. Prior to the experiment, the materials were tested in an off-line sentence acceptability task.^6^

Four experimental lists were created. The stimuli contained 40 target sentences (10 in each condition per list), 80 fillers, and an additional 6 sentences for practice. The fillers consisted of 40 grammatical and 40 ungrammatical sentences containing different agreement violations (i.e., gender, number or person agreement mismatches). The filler sentences were the same in each list. To ensure that participants were reading for comprehension, forty filler sentences were followed by a yes/no comprehension question. Stimuli were pseudo-randomized so that no more than two target sentences were presented consecutively.

### 3.3 Procedure

Participants were tested individually in separate soundproof booths. Participants read the sentences in a word-by-word, self-paced moving window task (Just et al., 1982) implemented on a desktop PC with the DMDX package (Forster and Forster, 2003). Stimuli were presented on a 17-inch screen in 18-point size and white ink on a black background. After giving informed consent, participants read the instructions. Each trial began with the entire target sentence displayed on the screen, with a dash (−) replacing each letter. As participants read, they pressed the spacebar to reveal the next word, at which point the previous word reverted to dashes. Participants were instructed to read at a natural pace and to ensure that they understood what they were reading so that they could respond accurately to comprehension questions. Reading times (in ms) were measured for each word from the time it appeared on the screen until the spacebar was pressed for the next word. The last word of a sentence was presented with a period. The comprehension question appeared after the last word of the sentence had been read. Participants responded by pressing a left or right button and could then move on to the next trial by pressing the spacebar. Participants were instructed to response as quickly and as accurately as possible. No feedback was provided. The experimental session was preceded by 6 practice trials to familiarize the participant with the procedure. The experimental session lasted approximately 35 min.

### 3.4 Data analysis

Analyses were conducted using mixed-effects models with crossed random effects for subjects and items (Baayen et al., 2008). Reading times were log-transformed to minimize skewness (Vasishth and Nicenboim, 2016). Models included sum coded (−0.5, 0.5) fixed main effects of verb (SC-vs. OC-verb) and grammaticality (grammatical vs. ungrammatical), and their interaction. Random intercepts for participant and item were included, with random slopes specified for verb and person. Linear mixed-effects models were fitted using buildmer (Voeten, 2023) in the statistical software R (v. 4.3.0). Buildmer uses lmer from the lme4 package (Bates et al., 2015) and allows for a systematic and replicable way of simplifying random effects structures and testing fixed effects. Buildmer starts by attempting to fit the maximal model; if it fails to converge, buildmer simplifies the random effects structure via backwards stepwise elimination. Once the maximally converging model has been identified, buildmer calculates value of *p* for all fixed effects based on Satterthwaite denominator degrees of freedom using the lmerTest package (Kuznetsova et al., 2020). We followed current best practices for automatic and reproducible statistical outlier detection (Thériault et al., 2023). After identifying the maximally converging model, outliers were identified using the check outliers function from the performance package (Lüdecke et al., 2023). This function uses a composite outlier score that applies multiple multivariate distance metrics.

For reasons of space, we will report the results at the critical word (i.e., word 3, the reflexive verb) and the two following words (words 4 and 5).

## 4 Results

Mean comprehension accuracy was above 85% for all participants (mean = 91.6, SD = 4.2, range = 87.5–100), indicating that participants were paying attention during the task. No participants were excluded due to poor accuracy.

**Figure 1:**
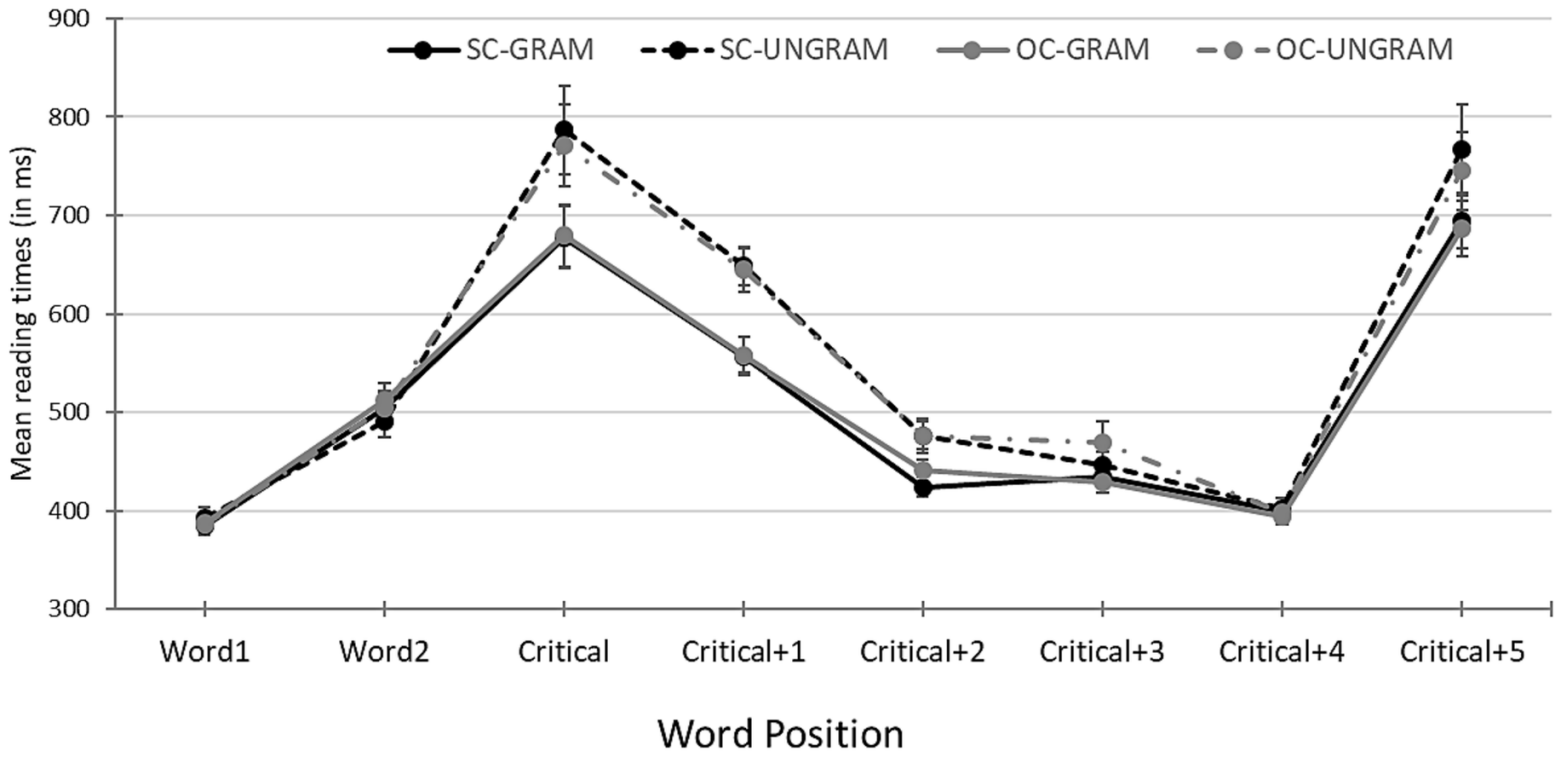
Word-by-word mean self-paced reading times segregated by verb type and grammaticality. Error bars represent the standard error of the mean computed across subject averages.

The results are illustrated in Figure 1. The results of the mixed-effects models for the critical word and the two following words appear in Table 2.

**Table 2:** Summary of linear mixed effects models (LMEM) fitted on log-transformed reading times at the critical word and the three following words.

	Estimate	SE	*t*-value	Slope	Value of *p*
Critical word					
Verb	0.002	0.024	0.104	(i)	0.917
Grammaticality	0.076	0.021	3.513	(p)	**< 0.001**
Verb * grammaticality	0.005	0.032	0.166		0.868
Critical word + 1					
Verb	−0.010	0.015	−0.661		0.508
Grammaticality	0.106	0.017	6.207	(p)	**< 0.001**
Verb * grammaticality	−0.007	0.031	−0.240		0.811
Critical Word + 2					
Verb	0.004	0.012	0.346		0.729
Grammaticality	0.058	0.015	3.832	(p)	**< 0.001**
Verb * grammaticality	−0.009	0.025	−0.365		0.714

Estimates, standard errors, *t*-values and *p*-values are reported for the main effects of Verb and grammaticality, as well as for the interaction of these two factors in each region. The “Slope” column indicates whether the model included the corresponding predictor as a random slope for participants (p), items (i) or both (p, i). Bold font indicates statistical significance.

The analysis revealed that at the critical word, the main effect of verb was not significant. At the critical word there was a significant main effect of grammaticality (estimate = 0.076, SE = 0.021, *t* = 3.513, *p* < 0.001), indicating that ungrammatical sentences produced longer reading times (778.8) than grammatical sentences (678.5). The interaction between the two factors was not significant. The analyses showed that at the two words following the critical one, the pattern of results was the same as in the critical word, that is, no main effect of verb, a significant main effect of grammaticality, and no interaction between verb and grammaticality.

## 5 Discussion

The aim of this study was to examine whether verb control information is rapidly used when assigning an antecedent to PRO. To this end, a self-paced reading study was conducted in which the control properties of the main verb, as well as grammaticality were manipulated. In the grammatical conditions, the reflexive pronoun agreed in person with the controller of PRO. In the ungrammatical conditions, the reflexive pronoun agreed in person with the non-controller of PRO, disagreeing in person with the controller of PRO. In such a manipulation, the critical word was the infinitive itself, and not a word following it. This manipulation makes it possible to examine whether control information is used as soon as the infinitive is processed.

Two theoretical proposals were contrasted. On the one hand, Frazier and colleagues (Frazier et al., 1983; Clifton and Frazier, 1986) suggest that verb control information is not used to initially assign an antecedent to PRO. Such an initial assignment is guided by a distance principle (i.e., the most recent filler strategy), which predicts that the less distant NP would be assigned as the antecedent of PRO. On the other hand, lexicalist parsing models (Boland et al., 1990; MacDonald et al., 1994; Trueswell and Tanenhaus, 1994, 1995; McRae and Matsuki, 2013) suggest that verb-specific information (such as control information) guides the initial assignment of an antecedent to PRO.

If the parser follows the most recent filler strategy, then we would expect an interaction between verb and grammaticality. This interaction would be driven by the fact that whereas for OC-sentences, ungrammatical items should produce longer reading times at the infinitive than grammatical items, for SC-sentences, grammatical items should produce longer reading times at the infinitive than ungrammatical items. In ungrammatical OC-items, the less distant NP and the reflexive pronoun did not agree in person, while in grammatical OC-items they agreed in person. For SC-items, there was a person clash between the less distant NP and the reflexive pronoun in grammatical, and not in ungrammatical sentences. In ungrammatical SC-items, the less distant NP and the reflexive pronoun agreed in person.

If the parser initially uses control information to coindex PRO with its controller, then we would expect a main effect of grammaticality, and no interaction between verb and grammaticality. More concretely, at the infinitive we would expect longer reading times for ungrammatical sentences than for grammatical sentences for both SC-and OC-items. Whereas in the ungrammatical items there was a person clash between the reflexive pronoun and the controller of PRO, in the grammatical conditions the reflexive pronoun and the controller agreed in person.

The results show that the main effect of verb as well as the interaction between verb and grammaticality were not significant at any of the three words analyzed. Critically, the results show that at the infinitive there was a significant main effect of grammaticality. When the reflexive pronoun agreed in person with the controller of PRO, reading times were significantly shorter than when the reflexive and the controller did not agree in person. The grammaticality effect was quite robust and long-lasting, as it ‘spills over’ to the two words following the infinitive verb.

The results presented here indicate that the parser rapidly uses information stored at a verb’s lexical entry. In order to detect the anomalies we have examined, the processor must access the information that specifies the control properties of the matrix verb. If the system were not using such information until a later stage, but following the most recent filler strategy, then one would expect increased reading times in response to reflexive pronouns that do not agree in person with the most recent NP (i.e., the object); that is, ungrammatical OC-items, and, most importantly, grammatical SC-items should produce larger reading times.

The finding that control information seems to have a similar effect in both SC-and OC-verbs supports semantic accounts of control phenomena (Jackendoff and Culicover, 2003; Culicover and Jackendoff, 2006). Such accounts define control as a lexical phenomenon that arises from specific semantic properties of verbs. According to the semantic account of control, no differences between SC-and OC-verbs would be expected, as reported in the present experiment. The fact that no differences were found between the two types of verbs goes against syntactic accounts of control (Rosenbaum, 1967; Hornstein, 1999; Chomsky and Lasnik, 2003). As discussed in the introduction, these accounts would predict significant differences between the two types of verbs, expecting a facilitation for OC sentences, since in these sentences the controller is closer to PRO, thus respecting the minimal distance principle and the minimal link condition.

The fact that no differences were found between SC-and OC-sentences is not consistent with the findings of Betancort et al. (2006), who reported a facilitation effect for OC-sentences in the region of NP2,^7^ which was attributed to a recency effect (i.e., proximity of antecedent and PRO). Surprisingly, this advantage for OC-over SC-items was not observed at the region of the infinitive, nor at the spillover region. In our view, this effect could be explained, not by the distance between PRO and its controller, but by the fact that in many items in Betancort et al. (2006) the NP2s in OC and SC were not lexically matched, since all NP2s in OC-items were preceded by the preposition “a” (i.e., “to”) whereas a significant number of NP2s in SC-items were preceded by other prepositions (e.g., *ante*, *con*, “in front of,” “with”) that are larger in terms of number of characters. Thus, the recency effect may be a length effect, rather than a reflection of a proximity effect between PRO and its controller. A recent eye-tracking study (De-Dios-Flores et al., 2019) that followed the same design as Betancort et al. but used lexically matched sentences between the two types of verbs showed no evidence for a recency effect.

One of the limitations of this study is the methodology used. Self-paced reading is not as time-sensitive as other techniques, such as eye-tracking. Unfortunately, due to coronavirus-related restrictions, the study could not be conducted by using the eye-tracking technique.

In summary, we have provided new data showing that subjects rapidly interpret the referential dependency between a phonologically null element and a lexically specified phrase, and, most remarkably, that control information has a very rapid influence on the process of selecting an antecedent for such a null subject. The data we have reported are congruent with theories of sentence processing (i.e., lexicalist parsing models; MacDonald et al., 1994; Trueswell and Tanenhaus, 1994, 1995; McRae and Matsuki, 2013) that assume the rapid influence of verb-specific information on the early stages of parsing.

## Data availability statement

The original contributions presented in the study are included in the article/supplementary material, further inquiries can be directed to the corresponding author.

## Ethics statement

The studies involving humans were approved by Ethics Committee on Research into People, Society and the Environment (CEIPSA-2021-PR-0024) of the Universitat Rovira i Virgili. The studies were conducted in accordance with the local legislation and institutional requirements. The participants provided their written informed consent to participate in this study.

## Author contributions

JD: Conceptualization, Data curation, Formal analysis, Funding acquisition, Investigation, Methodology, Writing – original draft, Writing – review & editing.

## Footnotes

^1^Small *pro* is a pronoun without phonetic properties that can be the subject of finite clauses in pro-drop languages, such as Italian and Spanish.

^2^Previous studies have examined the online processing of nominal control (e.g., Kwon and Sturt, 2014, 2016; Sturt and Kwon, 2015), adjunct control (e.g., Kwon and Sturt, 2014; Green et al., 2020) and implicit control (e.g., McCourt et al., 2015).

^3^The most recent filler strategy is reminiscent of Rosenbaum’s Minimal Distante Principle.

^4^Whereas Frazier et al. (1983) used sentences with a relative clause (“This is the girl the teacher decided to talk to”), Boland et al. (1990) used wh-questions (“Which grandson did the grandma attempt to guide toward the pony?”).

^5^ https://osf.io/p298g/

^6^ https://osf.io/p298g/

^7^First pass reading times in the region of NP2 were shorter in the OC condition than in the SC condition.
